# Lovastatin Potentiates the Function of α7-Nicotinic Acetylcholine Receptors

**DOI:** 10.3390/ph19060849

**Published:** 2026-05-29

**Authors:** Dmytro Isaev, Keun-Hang Susan Yang, Murat Oz

**Affiliations:** 1 Department of Cellular Membranology, Bogomoletz Institute of Physiology, 01024 Kiev, Ukraine; 2 Department of Biological Sciences, Schmid College of Science and Technology, Chapman University, One University Drive, Orange, CA 92866, USA; 3 Department of Pharmacology and Therapeutics, College of Pharmacy, Kuwait University, Safat 13110, Kuwait

**Keywords:** statins, lovastatin, nicotinic receptor, *Xenopus* oocyte

## Abstract

**Background/Objectives:** Statins are currently one of the most commonly used cholesterol-lowering drugs. In recent years, in addition to their well-known effects on the cardiovascular system, statins have been shown to exert beneficial effects in the progression of various neuropsychiatric and neurodegenerative diseases. **Methods:** In this study, the effects of lovastatin on the function of α7-nicotinic acetylcholine (nACh) receptors expressed in rat hippocampus and *Xenopus* oocytes were investigated. **Results:** In whole-cell patch clamp studies in hippocampal neurons, 21-day chronic (20 mg/kg), but not acute (20 min), lovastatin treatment caused significant potentiation of choline (a selective agonist for α7 nACh receptors)-induced currents and choline-induced increases in GABA_A_ receptor-mediated currents. Further studies in *Xenopus* oocytes expressing human α7-nACh receptors indicated that 72 h pretreatment with lovastatin caused a significant increase in α7-nACh receptor function with an EC_50_ value of 296 nM. Other statins, such as simvastatin and pravastatin, also potentiated α7-nACh receptors. Potentiation by lovastatin treatment was associated with a significant decrease in oocyte cholesterol content and was diminished by Go6983, an inhibitor of protein kinase C (PKC), suggesting that both decreased cholesterol levels and activation of PKC are involved in statin potentiation of α7-nACh receptors. **Conclusions:** In conclusion, our findings indicate that chronic lovastatin treatment potentiates the function of α7-nACh receptors in hippocampal neurons and in *Xenopus* oocytes expressing human α7-nACh receptors and provides important insights that could guide future efforts to design novel drugs targeting α7-nACh receptors.

## 1. Introduction

Statins, inhibitors of 3-hydroxy-3-methyl-glytarylcoenzyme A (HMG-CoA) reductase, are among the most effective and widely used cholesterol-lowering medications employed in treatment of hyperlipidemias and atherosclerotic cardiovascular diseases. Nearly 30% of adults 40 years and older in the United States were on a statin in 2014 [1]. Several clinical studies suggest that statins, in addition to lowering cholesterol, have beneficial pleiotropic effects in treatment of various neuropsychiatric and neurodegenerative diseases such as psychosis [2,3,4], Alzheimer [5,6] and Parkinson diseases [7,8]. However, mechanisms of pleiotropic effects of statins in these wide range of neuropsychiatric diseases are largely unknown.

Nicotinic acetylcholine (nACh) receptors, together with 5-hydroxytryptamine type3 (5-HT_3_), γ-aminobutyric acid subtype A (GABA_A_), and glycine receptors, are members of a structurally related family of pentameric ligand-gated ion channels [9,10]. The homomeric combination of α7-subunits of nAChRs form a rapidly activating and desensitizing pentameric ion channel–receptor complex. Importantly, these receptors exhibit high calcium permeability (PCa/PNa ≈ 10), implicating their role in neurotransmitter release. In addition, they are widely expressed on glutamatergic as well as the GABAergic nerve terminals, indicating that they can play important roles in presynaptic modulation of both excitatory and inhibitory neurotransmission in the hippocampus and other regions of the brain [9,11]. In the peripheric regions, α7-nACh receptors have been suggested to play important roles in inflammation and neuropathic pain [12,13,14]. In the central nervous system, these receptors have been shown to be involved in the pathogenesis of various neurodegenerative diseases such as Alzheimer and Parkinson diseases as well as neurodevelopmental disorders such as schizophrenia and autism [10,15,16,17,18]. In view of a significant overlap between the proposed pleiotropic effects of statins and pharmacological actions of α7-nACh receptor-modulating drugs in various neurological diseases, we hypothesized that lovastatin, a lipophilic statin, interacts with and modulates the function of α7-nACh receptors. To this end, we investigated the effects of lovastatin on the function of α7-nACh receptors in hippocampal neurons and *Xenopus* oocytes expressing human α7-nACh receptors.

## 2. Results

### 2.1. The Effect of Acute Lovastatin Treatment on α7-nACh Receptors in Hippocampal Slices

**Figure 1:**
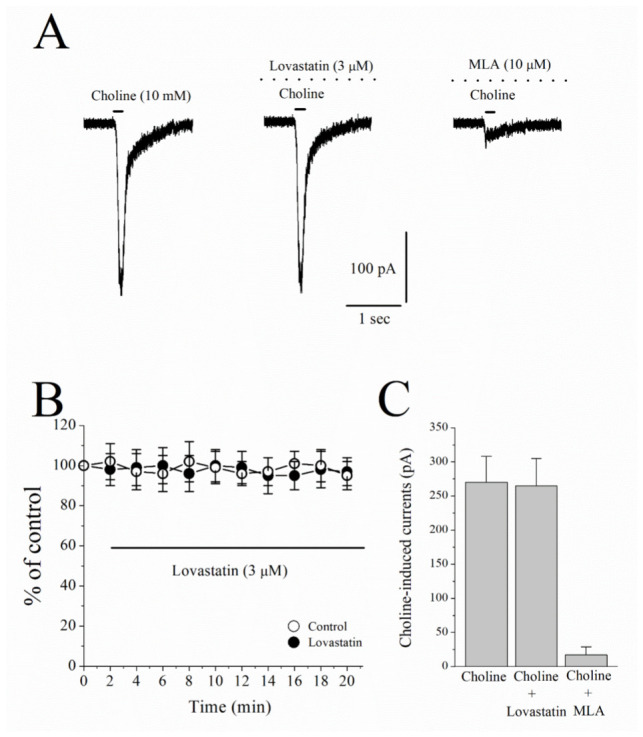
The effect of acute lovastatin administration on choline-induced ion currents recorded in CA1 area stratum radiatum interneurons of rat hippocampal slices. (**A**) Traces of choline (10 mM)-induced currents before (control, left panel) and after 20 min bath application of 3 µM lovastatin (in the middle). The effect of 1 min application of 10 µM methyllycaconitine (MLA, on the right). Choline application was represented with a short solid bar on top of the current traces. Dashed line indicates continuing bath application of lovastatin or MLA. (**B**) Time course of the effect of vehicle (0.1% DMSO; open circles) and lovastatin (3 µM; filled circles) on the peak amplitudes of choline-induced currents. Each data point represents the normalized mean ± S.E.M. of 6 to 7 cells. Duration of drug application is indicated by the horizontal bar in the figure. (**C**) Summary of the effects of lovastatin and methyllycaconitine on the peak amplitudes of choline-induced currents. Bars represent the means ± S.E.M of 5 to 8 experiments. MLA, methyllycaconitine.

In whole-cell patch clamp mode, focal application of 10 mM choline, a selective agonist for α7-nACh receptor, caused a rapidly activating and desensitizing inward currents that were completely inhibited by the bath application of 10 µM methyllycaconitine (MLA), a selective antagonist for α7-nACh receptor (Figure 1A; n = 5). Bath application of lovastatin (3 µM) for recordings lasting up to 20 min did not affect the maximal amplitudes of choline-induced currents (Figure 1A). Time course of the effects of lovastatin and the vehicle applications on the amplitudes of choline-induced currents is presented in Figure 1B (n = 6–7). In the absence of lovastatin, vehicle (0.1% DMSO) alone did not alter the amplitude of the choline-induced current (Figure 1B, controls). Summary of the acute effect of lovastatin and MLA was presented in Figure 1C.

### 2.2. The Effect of Chronic Lovastatin Treatment on α7-nACh Receptors in Hippocampal Slices

**Figure 2:**
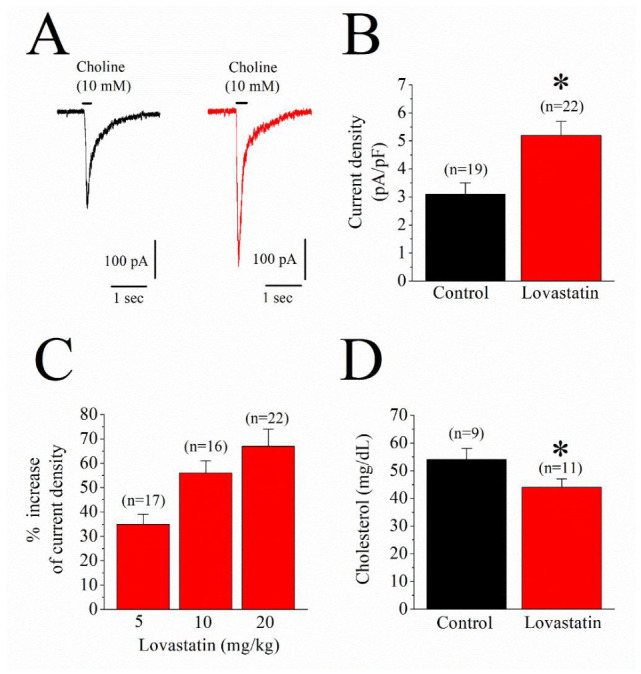
Effects of chronic lovastatin treatment on choline-induced ion currents recorded in CA1 area stratum radiatum interneurons of hippocampal slices. (**A**) Traces of choline-induced currents in vehicle-treated (control, on the left) and chronic (21-day, 20 mg/kg) lovastatin-treated (on the right, red color) hippocampal interneurons. Choline application (10 mM) was represented with a short solid bar on top of the current traces. (**B**) Summary of the effect of lovastatin treatment on the current densities of choline-activated responses. Averages of 3 consecutive current responses to 10 mM choline in each cell were calculated and the mean ± S.E. values were presented for each group. Bars represent the means ± S.E.M. of 19 to 22 cells (* *p* < 0.05 vs. control; ANOVA). (**C**) Concentration-dependent effect of chronic lovastatin treatment on choline-induced currents (n = 16–22). (**D**) The effect of chronic lovastatin treatment on serum cholesterol levels. Cholesterol levels were presented after 21-day vehicle or lovastatin treatment. Bars represent the means ± S.E.M of 9 to 11 cells (* * p* < 0.05 vs. control; ANOVA).

Considering the ineffectiveness of acute lovastatin application, we next tested the effect of 21-day chronic lovastatin (20 mg/kg, i.p.) treatment on the function of α7-nACh receptor. Compared to control (vehicle-treated) group, choline-induced current densities were significantly potentiated in lovastatin-administered group (Figure 2A,B). Cell membrane capacitance, cell input resistance, and resting membrane potentials in control group were 113 ± 8 pF (n = 19), 137 ± 9 MΩ (n = 19), and 62 ± 4 mV (n = 19), respectively. These values for lovastatin-treated group were 108 ± 7 pF (n = 22), 143 ± 11 MΩ (n = 22), and 64 ± 3 mV (n = 22), respectively. There were no statistically significant differences in any of these parameters between control and lovastatin groups (*p* > 0.05, ANOVA, n = 19–21). Changing the lovastatin doses (5, 10, and 20 mg/kg) enhanced the potentiation of choline-induced currents in a concentration-dependent manner (Figure 2C). In addition, measurements of serum cholesterol levels indicated that 21-day lovastatin treatment significantly decreased cholesterol levels compared to 21-day vehicle-treated group (Figure 2D).

**Figure 3:**
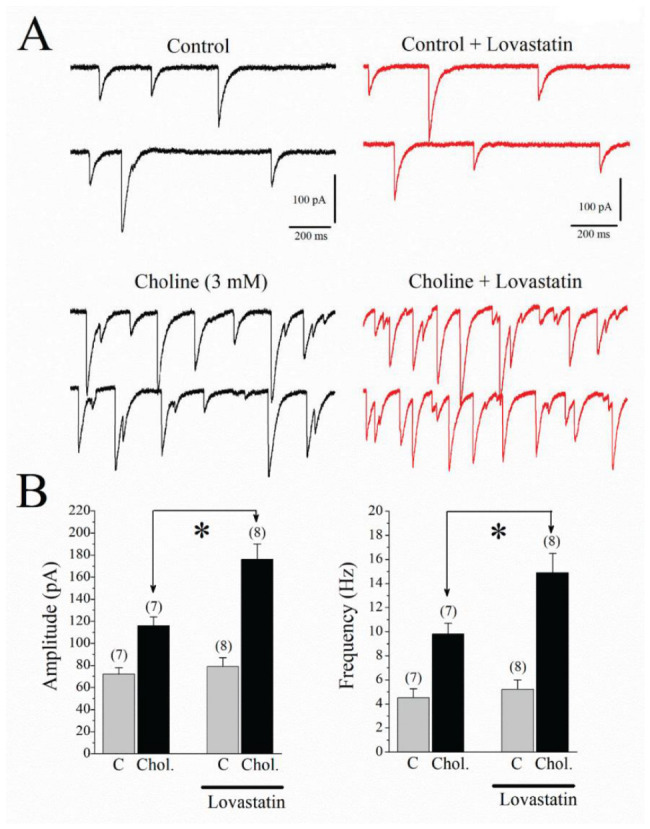
Effects of chronic lovastatin treatment on choline-induced enhancement of GABA_A_ receptor-mediated spontaneous synaptic events in CA1 pyramidal neurons. Whole-cell recordings were conducted using CsCl-based electrode solution at a holding potential of −70 mV. (**A**) On the left, application of choline (3 mM) for 10 s increased the amplitudes and frequencies of spontaneous inhibitory postsynaptic currents (sIPSCs; n = 7). On the right (in red color), choline-induced enhancements of sIPSCs were potentiated in chronic lovastatin (n =8)-treated neurons. (**B**) Summary of the effects of chronic lovastatin treatment on choline-induced responses. The averaged amplitudes (on the left) and the frequencies (on the right) of sIPSCs were presented before (control C, gray bars) and after (black bars) choline (Chol. 3 mM) application. For comparison, the effect of choline on the GABA_A_ receptor-mediated sIPSCs is shown in the vehicle-treated (control) and lovastatin-treated neurons. Bars represent the means ± S.E.M. of 7 to 8 experiments (* *p* < 0.05 vs. control; ANOVA). C, control; Chol., choline.

In the hippocampus, α7-nAChRs are located on both GABAergic and glutamatergic interneurons, and application of choline, a selective agonist for α7-nAChRs, increases both excitatory and inhibitory inputs to CA1 pyramidal neurons [11]. For this reason, we have isolated spontaneously occurring GABAA receptor-mediated synaptic currents, spontaneous inhibitory postsynaptic currents (sIPSCs) by pharmacological means (APV plus DNQX) and recorded choline-induced GABA responses in CA1 pyramidal neurons of hippocampal slices. Thereafter the effects of choline were assessed in the control and 21-day lovastatin-treated (20 mg/kg) groups. In agreement with our earlier studies [19,20], application of choline (3 mM for 15 s) caused a transient increase in amplitudes and frequencies of sIPSCs that lasted for 2–3 min (Figure 3A,B; n = 7–8). Choline-induced increases in sIPSCs were previously shown to be completely abolished by pre-application of MLA [19,20,21], indicating that the effect of choline was mediated by activation of the α7 nACh receptor. Comparing controls and 21-day lovastatin treatment group indicated that the effects of choline on the amplitudes and the frequencies of sIPSCs in pyramidal neurons were significantly potentiated in lovastatin-treated neurons (Figure 3B). When miniature IPSCs (mIPSCs) were measured in the presence of 1 µM TTX, the amplitudes and frequencies of mIPSCs in controls and lovastatin-treated group were 42.7 ± 3.8 pA and 0.9 ± 0.2 Hz (n = 5), and 46.3 ± 6.1 pA and 1.1 ± 0.3 Hz (n = 5), respectively. There were no statistically significant differences between control and lovastatin-treated groups with respect to means of amplitudes and frequencies (*p* > 0.05, ANOVA, n = 5), suggesting that postsynaptic GABA_A_ receptors are not affected by 21-day lovastatin treatment.

### 2.3. The Effect of Chronic Lovastatin Treatment in Xenopus Oocytes Expressing Human α7-nACh Receptor

**Figure 4:**
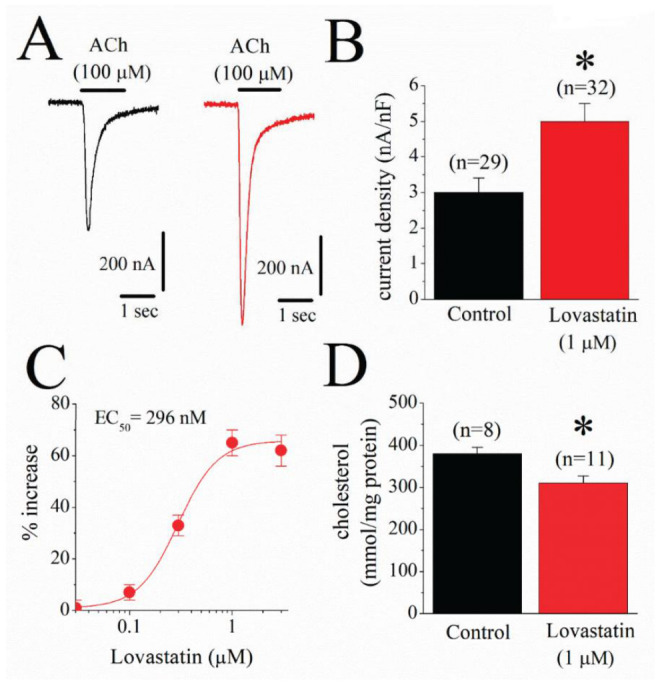
Effects of lovastatin treatment on the function of α7-nicotinic acetylcholine receptors expressed in *Xenopus* oocytes. (**A**) Traces of currents activated by 100 µM ACh in vehicle (on the left, black trace) or 72 h lovastatin (1 µM)-treated (on the right, red trace) oocytes. (**B**) Summary of the effects of 72 h lovastatin treatment on the density of ACh-induced currents (* *p* < 0.05 vs. control; ANOVA, n = 29–32). Averages of 3 consecutive current responses to 100 µM ACh were calculated and the mean ± S.E. values were presented for each group. (**C**) Concentration-dependent effect of chronic lovastatin administration. Increasing concentrations of lovastatin were administered for 72 h and % increase in ACh (100 µM)-induced currents (potentiation) compared to controls were presented. The mean ± S.E.M values were presented for each concentration group (n = 26–32). (**D**) Estimation of cholesterol content in control and lovastatin-treated *Xenopus* oocytes. Cholesterol content was measured in control (72 h in 0.1% *v*/*v* DMSO) and 72 h 1 µM lovastatin-treated oocytes. Bars represent the means ± S.E.M. of triplicate points from 8 to 11 experiments. * indicates *p* < 0.05 (ANOVA) compared to control group.

In *Xenopus* oocytes expressing human α7-nACh receptor, the effects of chronic (72 h) treatment with 1 µM lovastatin on ACh (100 µM)-induced currents were investigated. Compared to vehicle (0.1% DMSO)-treated oocytes, treatment with lovastatin caused a significant increase in the maximal amplitudes of ACh-induced currents (Figure 4A,B; *p* < 0.05, ANOVA, n = 29–32). Potentiating effect of lovastatin on α7-nACh receptors was concentration-dependent with an EC_50_ value of 296 nM (Figure 4C). Measurement of cholesterol levels indicated that, compared to vehicle-treated oocytes, 72 h treatment with 1 µM lovastatin caused a significant decrease in cholesterol content of oocytes (Figure 4D; *p* < 0.05, ANOVA, n = 8–11).

**Figure 5:**
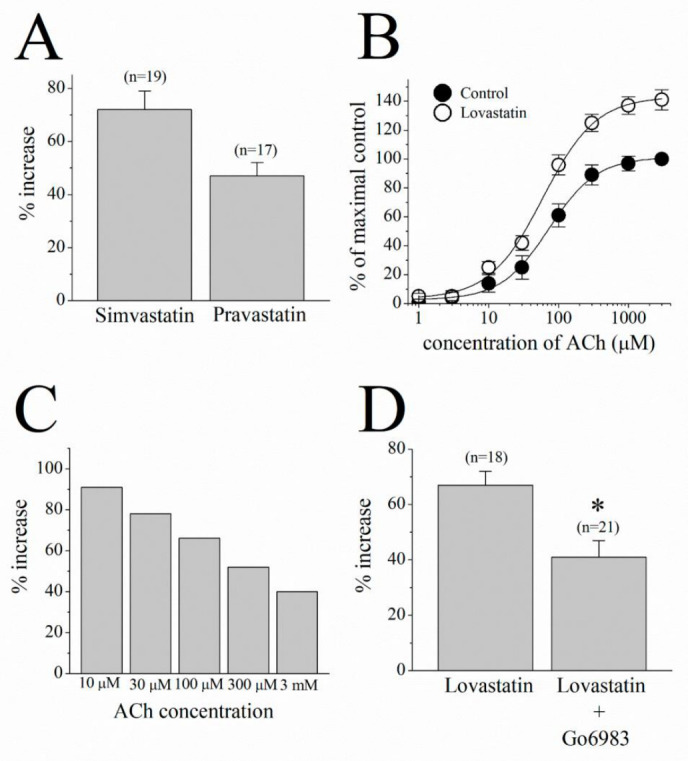
The effects of statins and Go6983 on α7-nACh receptor and on the acetylcholine concentration–response relationship. (**A**) Effect of 72 h pretreatment with 1 µM simvastatin or pravastatin on α7-nACh receptor-mediated currents. (**B**) Effect of 72 h pretreatment with 1 µM lovastatin on the acetylcholine (ACh) concentration–response relationship in *Xenopus* oocytes expressing human α7-nACh receptors. The filled and open circles represent controls and after lovastatin treatment, respectively. Each data point indicates the means ± S.E.M. of 12 to 14 experiments. (**C**) The effects of different ACh concentrations on the lovastatin potentiation of ACh-induced currents. Data were inferred directly from ACh concentration–response plot shown in Figure 5B by dividing lovastatin values to controls and therefore do not have SEM values. (**D**) Effect of Go6983 on lovastatin potentiation of α7-nACh receptor. Oocytes were pretreated with lovastatin (1 µM) alone or lovastatin and Go6983 (20 nM) for 72 h (* *p* < 0.05; ANOVA; n = 18–21).

Treatments (72 h) with other statins such as simvastatin (1 µM) and pravastatin (1 µM) also caused 71 ± 6% (n = 19) and 48 ± 5% (n = 17) potentiation of α7-nACh receptors, respectively (Figure 5A). In the next series of experiments, the effect of 72 h pretreatment with 1 µM lovastatin on the ACh concentration–response relationship was studied in *Xenopus* oocytes expressing α7-nACh receptors (Figure 5B). The EC_50_ values in control and lovastatin-treated groups were 71.1 ± 7.6 µM and 64.9 ± 8.2 µM, respectively (*p* > 0.05, ANOVA, n = 12–14). Apparent Hill coefficients for control and lovastatin-treated groups were 1.3 ± 0.2 and 1.1 ± 0.1, respectively. The extent of statin potentiation (inferred from concentration–response curve presented in Figure 5B) tends to decrease with increasing ACh concentration (Figure 5C). In earlier studies, activation of protein kinase C (PKC) has been shown to potentiate the function of α7-nACh receptors [22,23]. Therefore, we tested the effect of Go6983, a potent and selective inhibitor of PKC [24]. In the presence of 20 nM Go6983, the extent of lovastatin-induced potentiation of α7-nACh receptors was significantly diminished (Figure 5D, *p* < 0.05, ANOVA, n = 18–21).

## 3. Discussions

Our findings indicate that chronic, but not acute, treatment with lovastatin potentiates the function of α7-nACh receptors in hippocampal neurons. Further studies in *Xenopus* oocytes expressing human α7-nACh receptors showed that 72 h treatment with lovastatin increases α7-nACh receptor-mediated currents in concentration-dependent manner with an IC_50_ of 296 nM. The effect of lovastatin was not specific to lovastatin as other statins such as pravastatin and simvastatin also caused potentiation of α7-nACh receptors.

In earlier studies, treatments with lovastatin significantly increased the α7-nACh receptor expression and the α7-nACh receptor binding sites in a cholesterol-independent manner in SH-SY5Y and PC12 cells [25,26] and human HTB-15 astrocytes [27]. Similarly, chronic (14-day) treatment with low (0.1–1 µM) lovastatin augmented cell-surface expression of α7-nACh receptors in cultured hippocampal neurons [28]. In another study, statins such as mevastatin and lovastatin significantly reversed the amyloid β_1–40_ peptide inhibition of sympathetic α7-nAChR-mediated nitrergic neurogenic dilation in porcine basilar arteries and Ca^2+^ influxes in cultured superior cervical ganglion cells [29]. In addition, treatment with simvastatin in Aβ_25–35_-mice significantly reduced the death of neuronal cells in a α7-nAChR-dependent manner [30]. Similarly, simvastatin prevents Aβ_25–35_-impaired neurogenesis in hippocampal dentate gyrus through α7-nACh receptor-dependent PI3K-Akt pathway and increasing BDNF levels [31]. In another study, neurotoxic effects of amyloid β-peptide on cultured neuronal cells were reversed by lovastatin through increased expression of α7-nACh receptors [32]. In addition to reversing amyloid β- peptide related pathologies, statins improve conditional learning tasks [33], enhance spatial cognition and increase long-term potentiation in hippocampal CA1 area neurons by upregulating the expression of α7-nACh receptors [34]. Another study reported that atorvastatin treatment exerted antidepressant effect in depressive states which depended on α7-nAChR expression in the ventral hippocampus [35]. Collectively these in vitro and in vivo studies, in agreement with our findings, suggest that statins exert beneficial effects in various disease models by upregulating the expression of α7-nACh receptors.

There are also some studies suggesting that statins can modulate the function of α7-nACh receptors. In patch clamp studies, inhibition of α7-nACh receptors by acetylcholinesterase inhibitors such as physostigmine, neostigmine, and galantamine were completely reversed by concurrent application of statins such as mevastatin and lovastatin in rat superior cervical ganglion neurons [36]. Similar to our findings, the treatment of hippocampal slices with simvastatin for 2 h induced a dose-dependent increase in the amplitude of ACh-induced currents and the level of α7-nACh receptor protein on the cell membrane [37]. These results indicate that, in addition to upregulating α7-nACh receptor expression, statins can modulate the function of these receptors. Although majority of these studies demonstrated upregulation of α7-nACh receptor expression by statins, the effect of lovastatin on receptor expression was not examined in this study.

Although the exact mechanism of statin effect on α7-nACh receptors is currently unknown, upregulation of α7-nACh receptor expression [28,34], depletion of membrane cholesterol [28,38], increased membrane trafficking [28,37], and increased activities of PKC and CaM kinase II [37] have been proposed as potential mechanisms. In line with some of these proposed mechanisms, chronic lovastatin treatment significantly decreased cholesterol content of oocytes suggesting that diminished cholesterol levels may be involved in the effects of statins. Conversely, increasing the cholesterol to phospholipid ratios in *Xenopus* oocytes has been shown to cause significant inhibition of α7-nACh receptors [38]. Our results suggest that, in addition to cholesterol depletion, activation of PKC also plays a role in statin-induced potentiation of α7-nACh receptors in *Xenopus* oocytes. In agreement with our findings, activation of PKC has been shown to increase the function of α7-nACh receptors [22,23,37].

Interestingly, the extent of statin potentiation diminished with increasing ACh concentration, suggesting that increased receptor desensitization at high ACh concentrations tends to attenuate the effect of statin on the α7-nACh receptor. In contrast to statins, cholesterol has been shown to inhibit the function of α7-nACh receptor [38] mainly by stabilizing receptor desensitization [39,40]. Thus, the reversal of constitutive cholesterol inhibition by statins may be less effective at high ACh concentrations favoring receptor desensitization. In an earlier study, the co-application of mevastatin and myriocin was reported to change the desensitization kinetics of α7-nACh receptors [41]. However, in agreement with an earlier study [37], desensitization kinetics of ACh-induced currents were not significantly affected by lovastatin treatments in oocytes (Supplemental Figure S1). Of note, although maximal ACh responses were potentiated, the EC_50_ values remained unaltered by lovastatin treatment suggesting that the affinity of ACh for α7-nACh receptors was not affected by lovastatin.

Statins, widely used anti-cholesterol drugs, have been proposed to exert beneficial effects in treatment of various neuropsychiatric disorders such as psychosis and autism as well as neurodegenerative diseases such as Alzheimer and Parkinson diseases in both preclinical [25,27,29,31,32,35,42] and clinical studies [2,3,5,6,7,8]. Importantly, α7-nACh receptors have been suggested to play significant roles in the pathogenesis of these neurodegenerative [10,15,17] and neuropsychiatric [16,18] diseases in which statins have been proposed to have clinically positive effects. Furthermore, similar to statins, compounds that potentiate the functions of α7-nACh receptors (mainly positive allosteric modulators of these receptors) have also been suggested to alleviate pathological signs and symptoms in various animal models of these neuropsychiatric diseases [15,19,21,43,44]. Interestingly, in an earlier preclinical study, chronic administration of simvastatin during the withdrawal phase has been shown to reduce nicotine-seeking behavior which provides further evidence that statins can interact with nicotinic neurotransmission pathways in the central nervous system [45]. In conclusion, our findings indicate that statins upregulate the function of α7-nACh receptors in native neurons and in the heterologous *Xenopus* oocyte expression system and provide important insights that may guide future drug design efforts targeting α7-nACh receptors in the treatment of neuropsychiatric and neurodegenerative diseases.

## 4. Methods

### 4.1. Recordings from Hippocampal Slices

Male Sprague Dawley rats (8 to 10 weeks old; 250–350 g) were housed in a pathogen-free, standard plastic cages within the facility maintained at 22 ± 2 °C under a 12 h light/dark cycle. Standard rat chow and water were provided ad libitum. For chronic treatment, control group (n = 10) received vehicle (polyethylene glycol 200) intraperitoneally (i.p.), while the treatment groups (n = 6–10) were administered lovastatin (5–20 mg/kg, i.p.; dissolved in vehicle) for 21 days. Treatment dose and duration were determined based on previously published reports [46,47,48]. The volume of drugs administered to each animal was 2 mL/kg, adjusted to body weight.

At the end of treatment period, the animal was deeply anesthetized with pentobarbital (40 mg/kg, i.p.) and decapitated using a guillotine. The brain was rapidly removed, and horizontal slices (350–400 μm) prepared using a vibratome (Pelco, Redding, CA, USA), as described earlier [49,50]. The artificial cerebral spinal fluid (ACSF) for cutting and incubating slices contained: in mM: 124 NaCI, 2.5 KCI, 1.2 NaH_2_PO_4_, 2.5 MgSO_4_, 10 D-glucose, 1 CaCI_2_, and 25.9 NaHCO_3_, saturated with 95% O_2_/5% CO_2_. Following at least one-hour incubation, slices were transferred to a recording chamber where they were superfused at a rate of 5 mL/min with ACSF. Whole-cell patches from the somata of CA1 area interneurons were made under visual control using upright microscope (Nikon Eclipse E600 FN, Garden City, NY, USA) equipped with infrared differential interference contrast video microscopy system and 40× water immersion objective lens. Whole-cell patch-clamp recordings were made with pipettes pulled on a Flaming/Brown electrode puller (Sutter Instruments, Novato, CA, USA). Pipettes were typically 3–5 MΩ when filled with an internal solution that contains in mM: 140 Cs-MeSO_3_, 4 NaCI, 1 MgCI_2_, 0.2 EGTA, 10 HEPES, 2 MgATP, 0.3 Na_3_GTP (pH 7.3 using additional CsOH or KOH and volume adjusted to 285 mOsm). Cells were voltage clamped using an Axopatch 200B amplifier (Molecular Devices-Axon Instruments, San Jose, CA, USA) at a holding potential of −70 mV. A picospritzer (General Valve, Fairfield, NJ, USA) was used to apply choline (10 mM; for 200 msec) from pipettes identical to those used for whole-cell recording. Atropine (1 µM), a muscarinic receptor antagonist and TTX (1 µM), an inhibitor of voltage-gated Na^+^ channels, were routinely included in extracellular solution. Data were sampled at 10 kHz, filtered at 2 kHz, recorded using pClamp 10 software (Molecular Devices-Axon Instruments, San Jose, CA, USA) on a computer via a Digidata 1321 analog–digital converter, and analyzed using OriginPro v8.5 (OriginLab, Boston, MA, USA). Input resistance of the neurons was determined from the slope of the current–voltage relationships at the range of −50 to −80 mV, as described earlier [51]. All chemicals including lovastatin, choline, methyllycaconitine, and other reagents were purchased from Sigma (St. Louis, MO, USA).

Spontaneous inhibitory postsynaptic currents were recorded in CA1 pyramidal neurons, as described before [20]. Cells were voltage clamped at −70 mV using whole-cell electrodes containing (in mM): CsCl 125.0, HEPES 10.0, EGTA 1.0, CaCl_2_ 0.1, Mg^2+^-ATP 2.0, Na^+^-GTP 0.2, and the quaternary lidocaine derivative QX-314 2, pH 7.25. The ACSF (bath application) contained D-AP5 (50 µM), an NMDA receptor antagonist + NBQX (10 µM), AMPA/Kainate receptor antagonist. Data were analyzed with Clampfit software of pClamp version 10 (Molecular Devices-Axon Instruments, San Jose, CA, USA) and Origin v8.5 (Microcal Software, Northampton, MA, USA).

### 4.2. Recordings from Oocytes

Mature female *Xenopus laevis* frogs were purchased from Xenopus 1 (Dexter, MI, USA). The procedures followed in this study were in accordance with the Guide for the Care and Use of Laboratory Animals of the National Institutes of Health (Bethesda, MD) and approved by the Ethics Committee of Health Sciences Center of the Kuwait University (Protocol number: 4093). Clusters of oocytes were removed surgically under benzocaine (Sigma, St. Louis, MO, USA) local anesthesia (0.03% *w*/*v*) and individual oocytes were dissected away manually in a solution containing (in mM): NaCl, 88; KCl, 1; NaHCO_3_, 2.4; MgSO_4_, 0.8; and HEPES, 10 (pH 7.5), as described earlier [52]. Dissected oocytes were then stored for two to seven days in modified Barth’s solution (MBS) containing (in mM): NaCl, 88; KCl, 1; NaHCO_3_, 2.4; CaCl_2_, 2; MgSO_4_, 0.8; and HEPES, 10 (pH 7.5), supplemented with 2 mM, sodium pyruvate, 10,000 IU/L penicillin, 10 mg/L streptomycin, 50 mg/L gentamicin, and 0.5 mM theophylline. Briefly, oocytes were placed in a 0.2 mL recording chamber and superfused at a rate of 3–4 mL/min. The bathing solution contained (in mM): NaCl, 96; KCl, 2; CaCl_2_, 1.8; MgCl_2_,1 and HEPES 5 (pH 7.5). The cells were impaled with two glass microelectrodes filled with a 3 M KCl (0.5–2 MΩ). The oocytes were routinely voltage clamped at a holding potential of −70 mV using a GeneClamp-500 amplifier (Molecular Devices-Axon Instruments, San Jose, CA, USA) and current responses were recorded and stored digitally for further analysis. Oocyte capacitance was measured by a paired-ramp method described earlier [53].

Drugs were applied by gravity flow via a micropipette positioned about 2 mm from the oocyte. Some of the compounds were applied externally by addition to the superfusate (flow rate of 3–4 mL/min). Acetylcholine, lovastatin (sodium salt), Go6983, and all chemicals used were obtained from Sigma (St. Louis, MO, USA). Stock solutions of lovastatin were prepared in DMSO at a concentration of 10 mM. At the highest final concentrations used (0.3% *v*/*v*), DMSO did not have a significant effect on ACh (100 µM)-induced currents (n = 4).

The cDNA clone of human α7-nACh receptor was employed to produce cRNA using in vitro transcription method. Capped cRNA transcripts were synthesized in vitro using a mMESSAGE mMACHINE kit from Ambion (Austin, TX, USA) and analyzed on 1.2% formaldehyde agarose gel to check the size and the quality of the transcripts. Using Nanoject III (Drummond Scientific Company, Broomall, PA, USA) microinjector, approximately 5 ng of cRNA was injected into each oocyte.

### 4.3. Cholesterol Measurements

Blood samples were collected from pentobarbital (40 mg/kg, i.p.) anesthetized rats via cardiac puncture using sterile syringes. Whole blood was transferred to plain 1.5 mL microcentrifuge tubes and allowed to clot at room temperature for 30 min, followed by centrifugation at 2000–3000× *g* for 10 min at 4 °C. The resulting serum supernatant was aspirated, aliquoted into labeled tubes and stored at −80 °C until biochemical analyses were performed. Cholesterol quantification assay kit (Cat, no: CS0005; Sigma-Aldrich, St. Louis, MO, USA) was used to measure serum cholesterol following the manual’s instructions.

For cholesterol measurements in oocytes, 100–150 oocytes per assay were suspended (approximately 20 μL/oocyte) in a homogenization buffer containing (in mM) HEPES 10, EDTA 1, PMSF 0.1, and 0.02% NaN3, 50 μg/mL bacitracin (pH 7.4) at 4 °C on ice and homogenized using a motorized Teflon homogenizer (six strokes, 15 s each at high speed) and sonicated (Soniprep 150; Sanyo, Moriguchi, Japan) for 1 min and stored at −20°C until needed. Cholesterol content was determined by the Amplex Red Cholesterol Assay Kit according to manufacturer’s instructions (Cat. no: A12216; Molecular Probes, Eugene, OR, USA). Protein concentration was determined using the Bicinchoninic acid assay (BCA) protein assay kit (Pierce, Rockford, IL, USA). Subsequently the concentration of cholesterol was normalized to protein concentration.

### 4.4. Statistical Analysis

Results of experiments are presented as the mean ± standard error means (S.E.M.). Paired *t*-tests, Student’s *t*-tests, or analysis of variance (ANOVA), followed by post hoc Bonferroni tests, were used to statistically evaluate the data. The Origin^TM^ (Microcal Software, Version 8.5, OriginLab Corporation, Northampton, MA, USA) software was used for data analysis and calculations. Logistic equation and non-linear decay or linear curve-fitting functions of the software were employed for data analysis.

## Data Availability

The data that support the findings of this study are available on request from the corresponding author The original contributions presented in this study are included in the article/supplementary materials. Further inquiries can be directed to the corresponding author.

## Supplementary Materials

The following supporting information can be downloaded at: https://www.mdpi.com/article/10.3390/ph19060849/s1, Figure S1: The effect of chronic lovastatin administration on the desensitization of α7-nACh receptors. Current traces normalized to maximal amplitudes and overlapped for comparison (on the left). Summary of the results comparing the averages of desensitization time constants (τ) between control (0.1% *v*/*v* DMSO) and 72 h lovastatin (1 µM) groups (on the right). There is no statistically significant difference between the groups (*p* > 0.05; ANOVA; n = 7–8).
