# Supplementary material for: Lovastatin Potentiates the Function of α7-Nicotinic Acetylcholine Receptors
Source: Pharmaceuticals (Basel). 2026 May 29;19(6):849. doi: 10.3390/ph19060849 (PMC13304601; doi:10.3390/ph19060849)
Supplement: Supplementary file 1 [file pharmaceuticals-19-00849-s001.zip › pharmaceuticals-4314592-supplementary.pdf]

Supplemantary figure 1

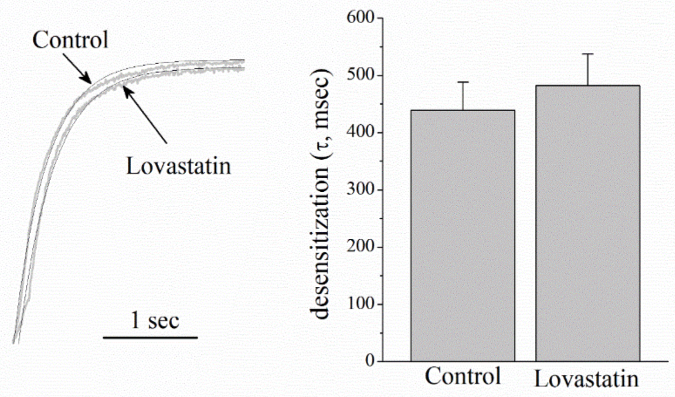

Supplemental Fgure S1. The effect of chronic lovastatin administration on the desensitization of α7-nACh receptors. Current traces normalized to maximal amplitudes and overlapped for comparison (on the left). Summary of the results comparing the averages of desensitization time constants (τ) between control (0.1% v/v DMSO) and 72-hour lovastatin (1 μM) groups (on the right). There is no statistically significant difference between the groups ($P>0.05$; ANOVA; n=7-8).
